# Prophylactic cranial irradiation in small cell lung cancer: a systematic review and meta-analysis

**DOI:** 10.1186/s12885-018-5251-3

**Published:** 2019-01-21

**Authors:** Xin Yin, Danfang Yan, Miao Qiu, Liming Huang, Sen-Xiang Yan

**Affiliations:** 1 Department of Radiation Oncology, the First Affiliated Hospital, College of Medicine, Zhejiang University, 79 Qingchun Road, Hangzhou Zhejiang, 310003 People’s Republic of China; 2 The First Department of Chemotherapy, The First Affiliated Hospital, Fujian Medical University, Fuzhou, Fujian 350005 People’s Republic of China

**Keywords:** Small-cell lung cancer, Prophylactic cranial irradiation, Brain metastasis

## Abstract

**Background:**

The efficacy of prophylactic cranial irradiation (PCI) in treating patients with small cell lung cancer (SCLC) has not been clear, and recent randomized studies have demonstrated conflicting results from previously published findings. The purpose of this study was to reevaluate the efficacy of PCI in patients with SCLC and to assess factors associated with its efficacy.

**Methods:**

We conducted a quantitative meta-analysis to explore the efficacy of PCI in patients with SCLC. A literature search was performed using EMBASE, MEDLINE, Cochrane and ClinicalTrials.gov databases. We pooled the data and compared overall survival (OS) and brain metastasis (BM) between patients treated with PCI (PCI group) and patients without PCI treatment (observation group).

**Results:**

Of the 1074 studies identified in our analysis, we selected seven studies including 2114 patients for the current meta-analysis. Our results showed that the PCI group showed decreased BM (HR = 0.45, 95% CI: 0.38–0.55, *P* < 0.001) and prolonged OS (HR = 0.81, 95% CI: 0.67–0.99, *P* < 0.001). However, in terms of OS, the pooled analysis showed a high heterogeneity (I^2^ = 74.1%, *P* = 0.001). In subgroup analyses of OS, we found that the heterogeneity mainly came from patients with brain imaging after initial chemoradiotherapy (HR = 0.94, 95% CI: 0.74–1.18, *P* = 0.59).

**Conclusions:**

The results of this study showed that PCI has a significant effect on decreasing BM but little benefit in prolonging OS when brain imaging was introduced to confirm lack of BM after initial chemoradiotherapy and before irradiation.

**Electronic supplementary material:**

The online version of this article (10.1186/s12885-018-5251-3) contains supplementary material, which is available to authorized users.

## Background

Small cell lung cancer (SCLC) accounts for approximately 15% of lung cancer and is known for its rapid doubling time and potential for widespread metastases [1, 2]. For the past several decades, the chemotherapeutic standard of care, cisplatin or carboplatin plus etoposide, has remained essentially unaltered for the treatment of SCLC [3–5]. With the current combination treatment of chemoradiotherapy (CRT), the risk of thoracic recurrence decreases, and as a result, brain metastasis (BM) becomes one of the main types of relapse [6]. Although patients initially respond well to CRT, the 2-year cumulative risk of developing BM is more than 50% and the median survival time after BM is only 4–5 months. Approximately 65% of patients have detectable BM on autopsy [7, 8].

Because the blood brain barrier restricts the penetration of most chemotherapeutic agents into the brain, leaving the brain as a susceptible site for relapse, prophylactic cranial irradiation (PCI) has been used for patients with SCLC [9]. A meta-analysis of studies that mainly included patients with limited stage SCLC (LS-SCLC) [10] showed that PCI not only decreased the incidence of BM but also prolonged overall survival (OS). Since then, two more trials have focused on PCI in extensive stage SCLC (ES-SCLC) [11, 12]. The European Organization for Research and Treatment of Cancer (EORTC) trial in 2007 found that PCI also decreased the risk of BM and prolonged OS in ES-SCLC [11]. However, although the findings of this study led to changes in guidelines and clinical practice, the effect of PCI was still subject to debate, as the patients in the EORTC study did not undergo routine brain MRI. Another randomized trial in patients with ES-SCLC was performed and brain MRI was performed on every patient after initial CRT [12]. The outcome showed that PCI had no benefit in prolonging OS in patients with ES-SCLC. Thus, in the latest version of NCCN guidelines (V3.2017), PCI has a lower recommendation level (category 2A) in ES-SCLC compared with LS-SCLC (category 1) [13]. The necessity of PCI remains controversial.

The aim of the present study was to reassess the effect of PCI in SCLC by performing a systematic review of randomized controlled trials (RCTs) published in the literature over the past 30 years.

## Methods

### Study protocol

A study protocol was drafted following the Cochrane Collaboration format [14].

### Eligibility criteria

The present systematic review only included studies that met the following criteria: 1) study type: RCT; 2) language restriction: English only; 3) participants: adult patients with cytologically or histologically confirmed SCLC in any response with no evidence of BM and without previous cranial irradiation; and 4) intervention: PCI. The exclusion criteria were as follows: 1) case control study, cohort study and retrospective study; 2) withdrawal rate: > 20%; and 3) participants: previous cranial irradiation history, < 18 years.

### Search strategy and information sources

Two of the authors (XY and DFY) independently searched the MEDLINE database for studies from Jan 1, 1987 up to June 1, 2017 using the combination of the variables “prophylactic cranial irradiation” AND “small cell lung cancer”. The search strategy for EMBASE and the Cochrane Library were similar to that used for MEDLINE. The search was limited to clinical studies. To ensure that all relevant studies had been included in this systematic review, reference lists from RCTs and systematic reviews were manually screened.

### Study selection and data collection

Two reviewers independently read titles, abstracts and full text papers and extracted data from the included full text papers. Discrepancies were resolved by a third referee. We only included RCTs that recruited patients with SCLC and assessed the efficacy and safety of PCI compared with observation. All patients were recruited with no evidence of BM and no previous cranial irradiation. After reading all included RCT articles, the following data were extracted from the included RCTs after strict selection and evaluation: basic information on the included trials, eligibility criteria and study design, and outcome assessments.

### Outcome definition and quality assessment

The included outcomes were defined as follows: OS, defined as the time from randomization until death due to any cause; and BM, defined as the incidence of BM diagnosed on the basis of either brain MRI or CT or symptoms suggestive of BM. Two authors (XY and DFY) independently assessed for methodological quality using the Jadad scale [15], which includes the following four items: randomization, allocation concealment, blinding of participants and personnel, and withdrawals and dropouts. The range of scores is from 0 to 7. Any study with a Jadad score below 3 was considered to be of poor quality. The possibility of publication bias across studies was assessed using funnel plots and Begg’s test.

### Statistical analyses

The OS and BM outcomes were measured in terms of the hazard ratio (HR) of the PCI group compared with the observation group. For each trial, the HR with the 95% confidence interval (95% CI) was directly extracted from the research article or calculated using other available statistical information by two independent reviewers according to the method provided by Tierney [16]. An HR < 1 implied a survival benefit for the PCI arm. Statistical heterogeneity was estimated by the I^2^ statistic [17]. A random-effects model was used if I^2^ > 50%, and a fixed-effects model if I^2^ < 50%. To investigate the sources of heterogeneity, predefined subgroup analyses were performed: the extent of disease (limited vs. extensive stage), brain imaging after initial CRT (brain imaging vs. no brain imaging) and response to initial chemotherapy (complete response vs. any response). Tests were two-tailed and a *P* value of less than 0.05 was considered to be significant for all analyses. All data analyses were performed using Review Manager (RevMan) version 5.3 and Stata 12.0.

## Results

### Study selection and characteristics

**Fig. 1:**
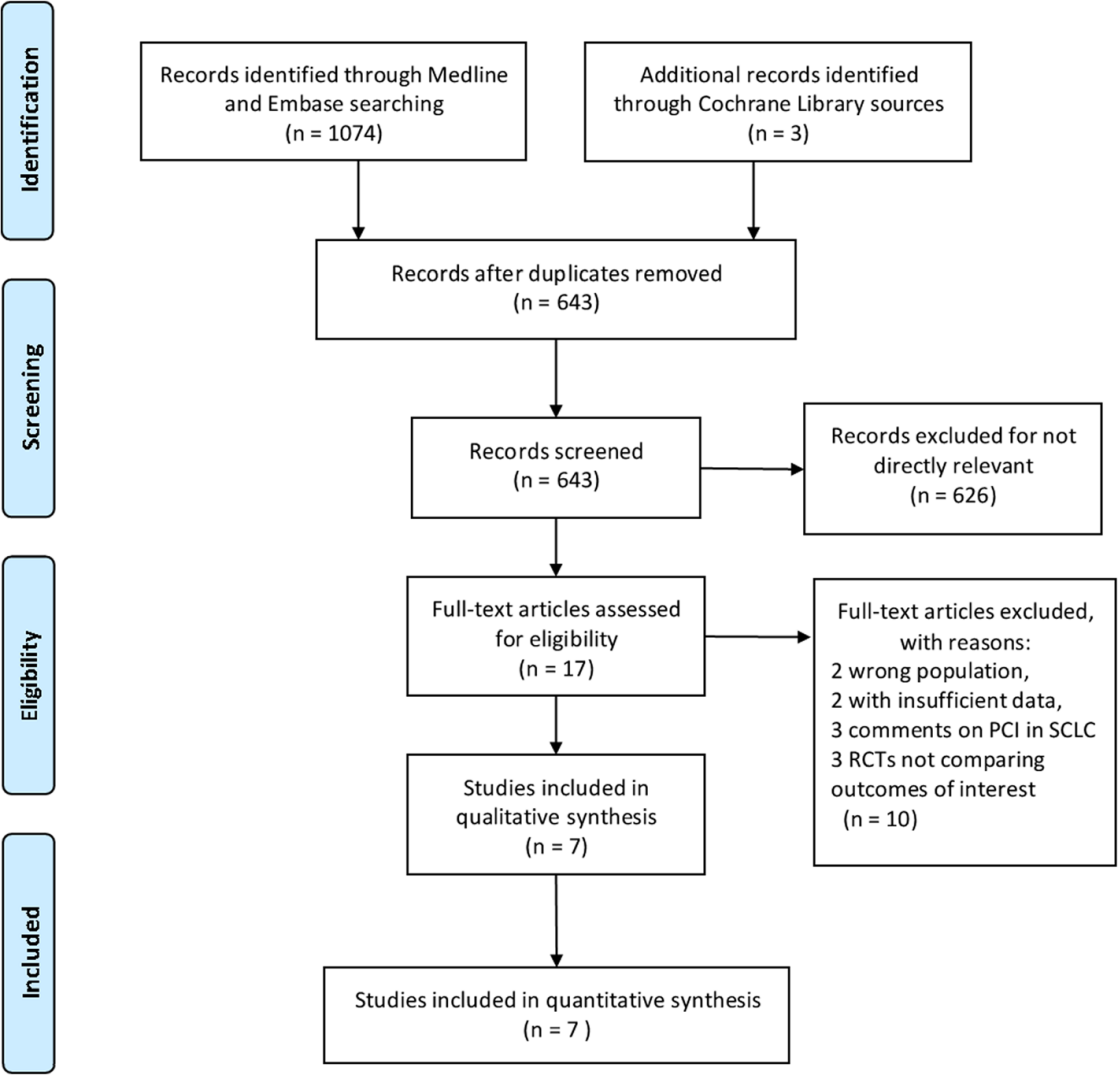
PRISMA flow diagram of study selection. RCT: randomized controlled trial

A total of 1074 titles and abstracts were screened (Fig. 1). After removing the duplicates and irrelevant records, 17 full-text articles were assessed for eligibility. An additional 10 articles were excluded due to the limitation of publication types: two with the wrong population, two with insufficient data, three comments on PCI in SCLC and three RCTs not comparing outcomes of interest. Ultimately, a total of 7 RCTs [11, 12, 18–22] published between January 1, 1987 and June 6, 2017 met the eligibility criteria for this meta-analysis. These trials enrolled a total of 2114 patients with SCLC and all patients were enrolled after 1980. One trial [18] was of small sample size, with 46 patients. The remaining six trials each had more than 200 patients. The average age of all the patients ranged from 56 to 69 years. Male patients accounted for 57–89% and 57–92% in the PCI and observation groups, respectively, but there was a good gender match between each trial. The dose of cranial irradiation ranged from 24 to 40 Gy. Two trials [11, 12] only enrolled patients at extensive stage and one trial [20] only at limited stage. The remaining four trials [18, 19, 21, 22] included both stages. Four trials [18–21] compared the efficacy of PCI in patients with complete response after initial chemotherapy. The other three trials [11, 12, 22] did not limit the response state. Brain imaging was performed before randomization in four studies [12, 18, 19, 21]. Of the 7 studies included in the analysis, all were single trial analysis except one [22], which was a pooled analysis of four trials. The detailed characteristics of the included studies are listed in Table 1.

**Table 1:** Summary of the studies included in the systematic review and meta-analysis

Study	Age		Gender (male %)		Brain scan	Complete Response (%)		Extent of disease				Median FU (year)	PCI schedule (Gy/fraction)	N. of patients
								PCI		No PCI				
	PCI	No PCI	PCI	No PCI		PCI	No PCI	LD %	ED %	LD %	ED %			
Takahashi et al., 2017	69 (43–83)	69 (37–86)	84	88	yes	15	14	0	100	0	100	1	25/10	224
Schild et al., 2012	63 (34–79)	63 (37–80)	57	60	no	NC	NC	73	27	31	69	6	30/15; 25/10	739
Slotman et al., 2007	63 (37–75)	63 (39–75)	68	57	no	NC	NC	0	100	0	100	0.8	20–30/5–12	286
Laplanche et al., 1998	58 ± 8	57 ± 9	89	92	yes	100		83	17	86	14	5	24–30/8–15	211
Gregor et al., 1997	60 (37–79)	61 (28–76)	64	62	no	100		100	0	100	0	42	8–38/1–18	314
Arriagada et al., 1995	56 ± 9	57 ± 8	88	86	yes	100		83	17	79	21	8.3	24/8	294
Ohonoshi et al., 1993	62 (43–75)	66 (45–79)	65	83	yes	100		61	39	70	30	7.5	40/20	46

*PCI* prophylactic cranial irradiation, *FU* follow up, *N* number, *NC* not clear, *LD* limited disease, *ED* extensive disease

### Quality assessment and publication Bias

Generally, the included studies had high quality. Three studies [11, 12, 21] scored a Jadad score of 5 and four studies [18–20, 22] scored a Jadad score of 4. No study scored below a 3, which was considered as low quality. Details about the score of each included study are shown in Additional file 1: Table S1. Funnel plots and Begg’s test results are shown in Additional file 2: Figure S1. We did not find statistically significant asymmetry to indicate publication bias (all *P* values > 0.5).

### Efficacy outcomes

All 7 RCTs were available for the analysis of OS outcomes. The RCTs included a total of 2114 patients. For BM outcomes, only one trial [22] was not available for analyses, because of lack of data.

### OS outcome

**Fig. 2:**
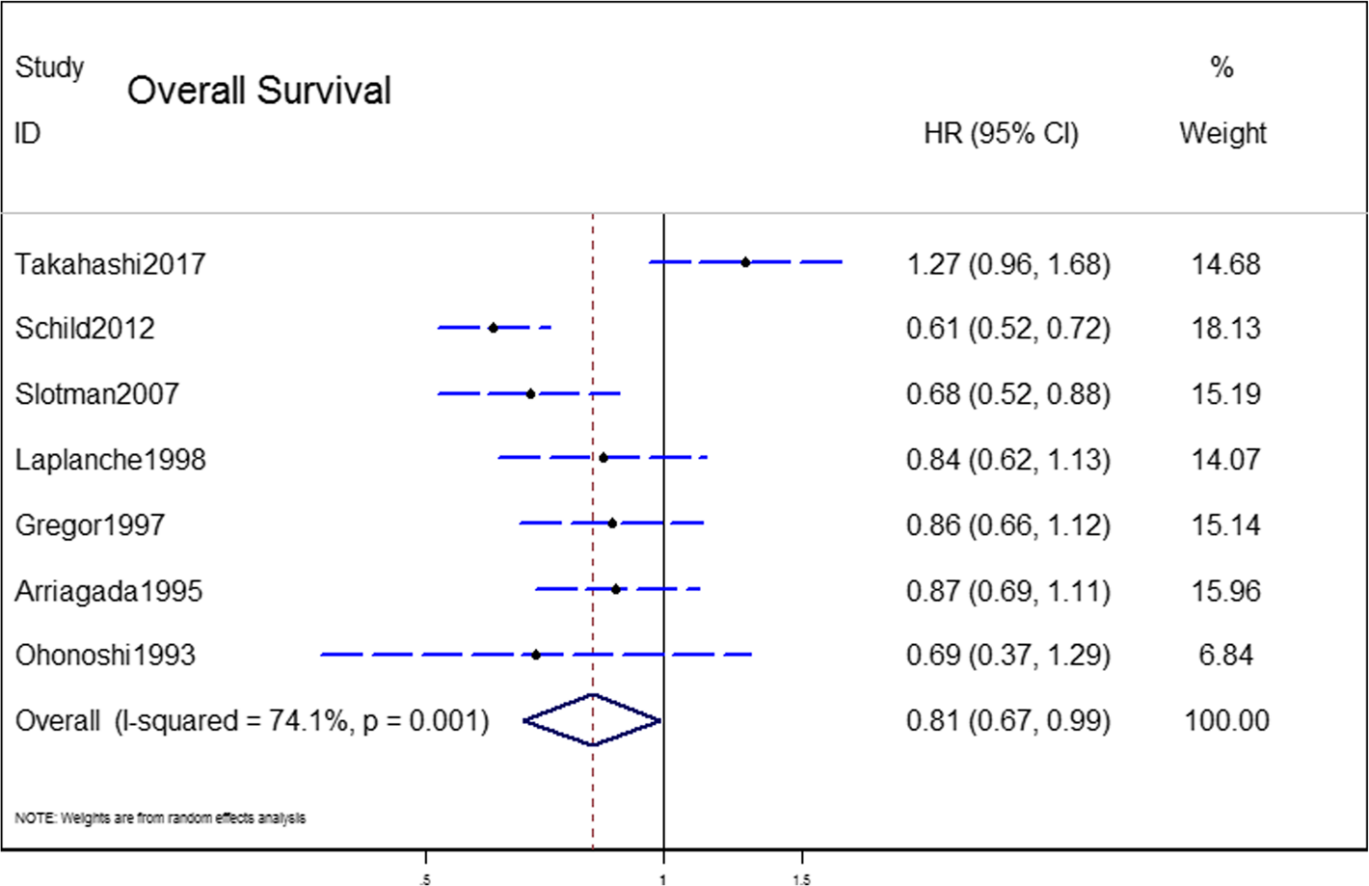
Results of the meta-analysis of the studies evaluating the role of PCI on overall survival (OS). Hazard ratio (HR): 0.81 (95% CI: 0.67–0.99)

The OS outcome was measured in terms of the HR of undergoing PCI compared with observation. The pooled model showed a survival benefit with PCI than with observation in the patients with SCLC (HR = 0.81, 95% CI: 0.67–0.99, *P* < 0.001) (Fig. 2). A significant statistical heterogeneity was noted in this analysis (I^2^ = 74.1%, *P* = 0.001), which was largely attributable to the recently published trial [12] that showed a different direction of effect (HR = 1.27) from the other 6 trials (HRs from 0.59–0.87). Thus, we combined the results using a random-effect model.

### BM outcome

**Fig. 3:**
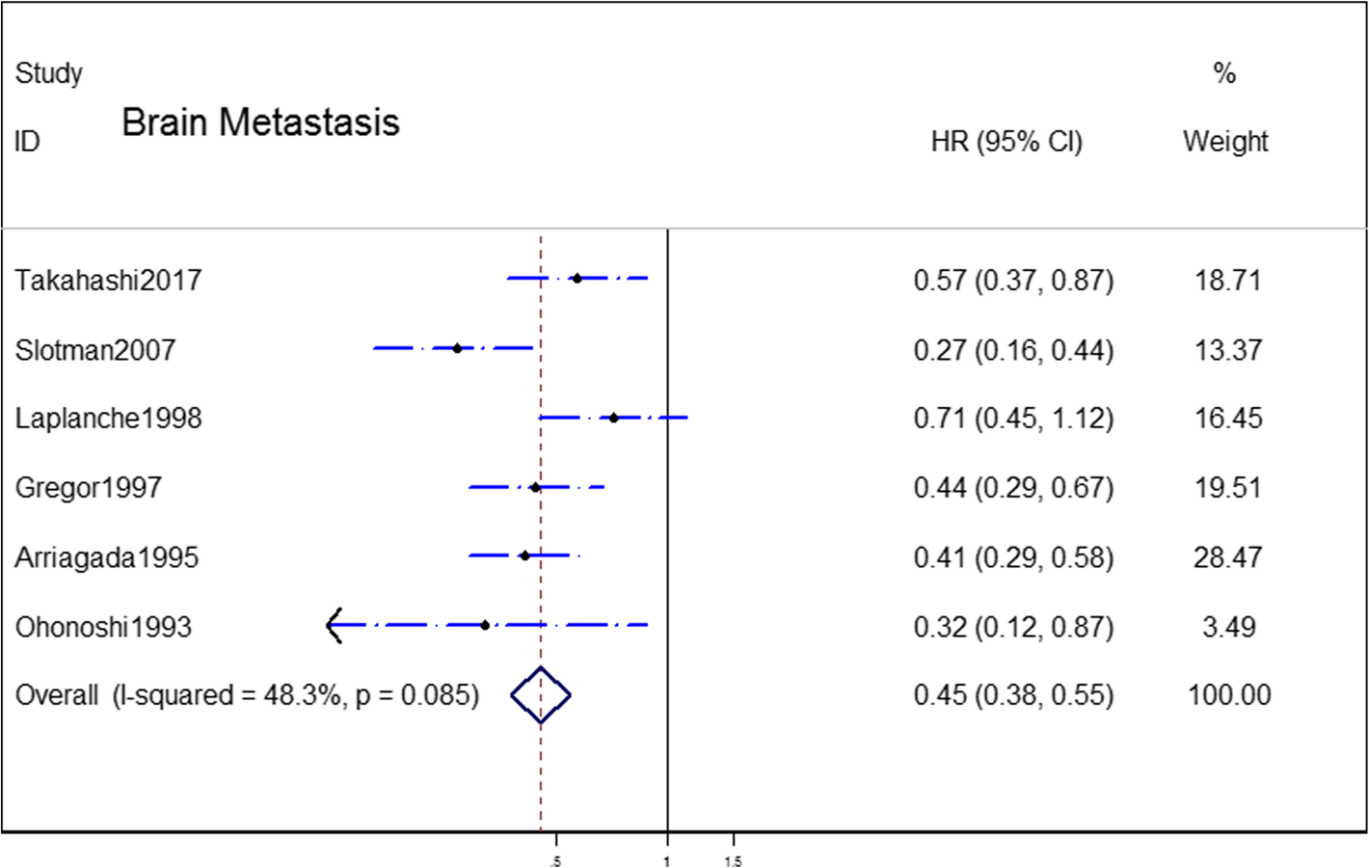
Results of the meta-analysis of the studies evaluating the role of PCI on brain metastases (BM). Hazard ratio (HR): 0.45 (95% CI: 0.38–0.55)

The combined result revealed a great decrease in BM in the group assigned to PCI compared with the observation group, with a pooled HR of 0.45 (95% CI = 0.38–0.55) (Fig. 3). The heterogeneity between the PCI group and observation group was not statistically significant (I^2^ = 48.3%, *P* = 0.08). Thus, we combined the results by a fixed-effect model.

### Subgroup analyses

**Fig. 4:**
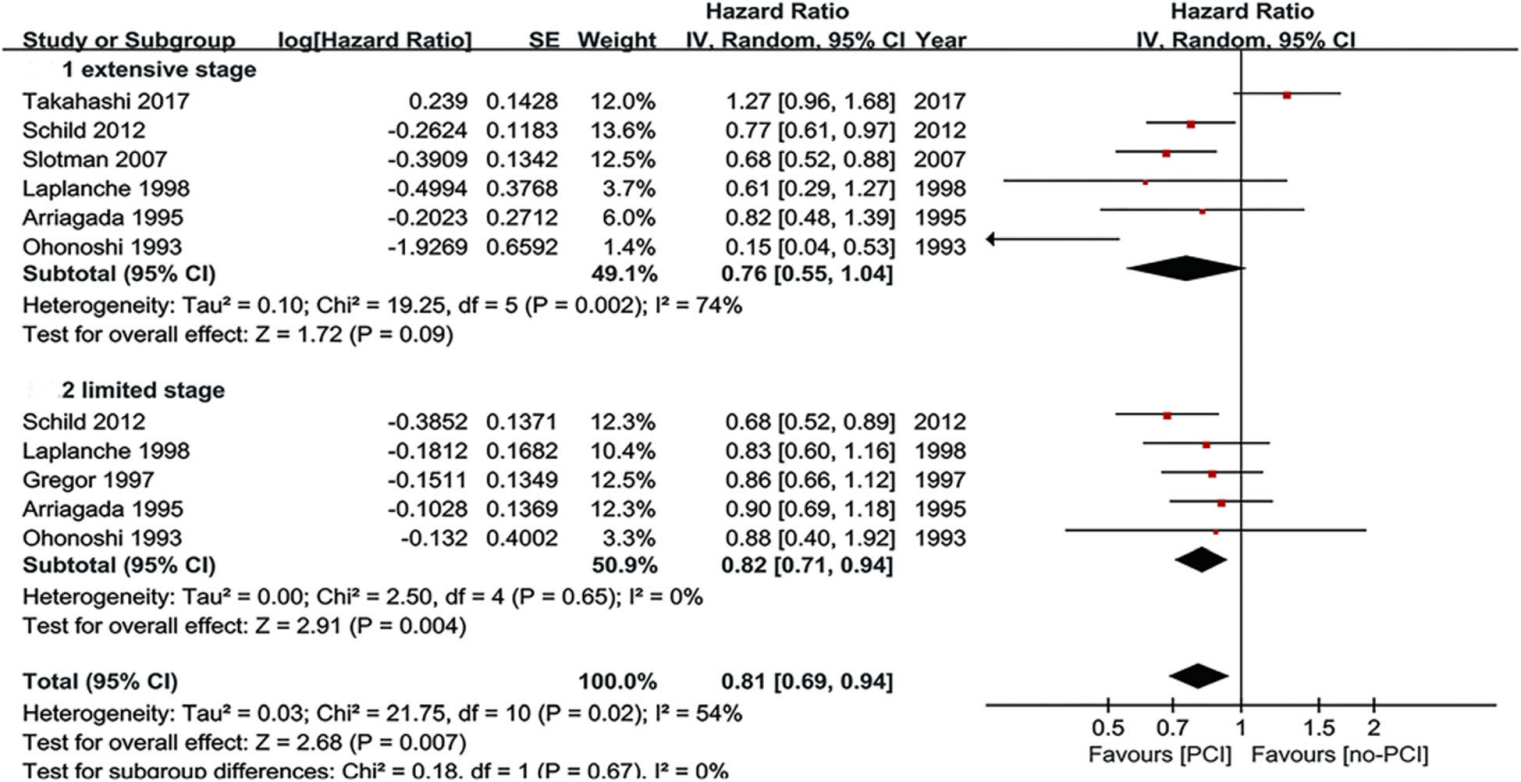
Subgroup analysis for OS according to stage of disease

**Fig. 5:**
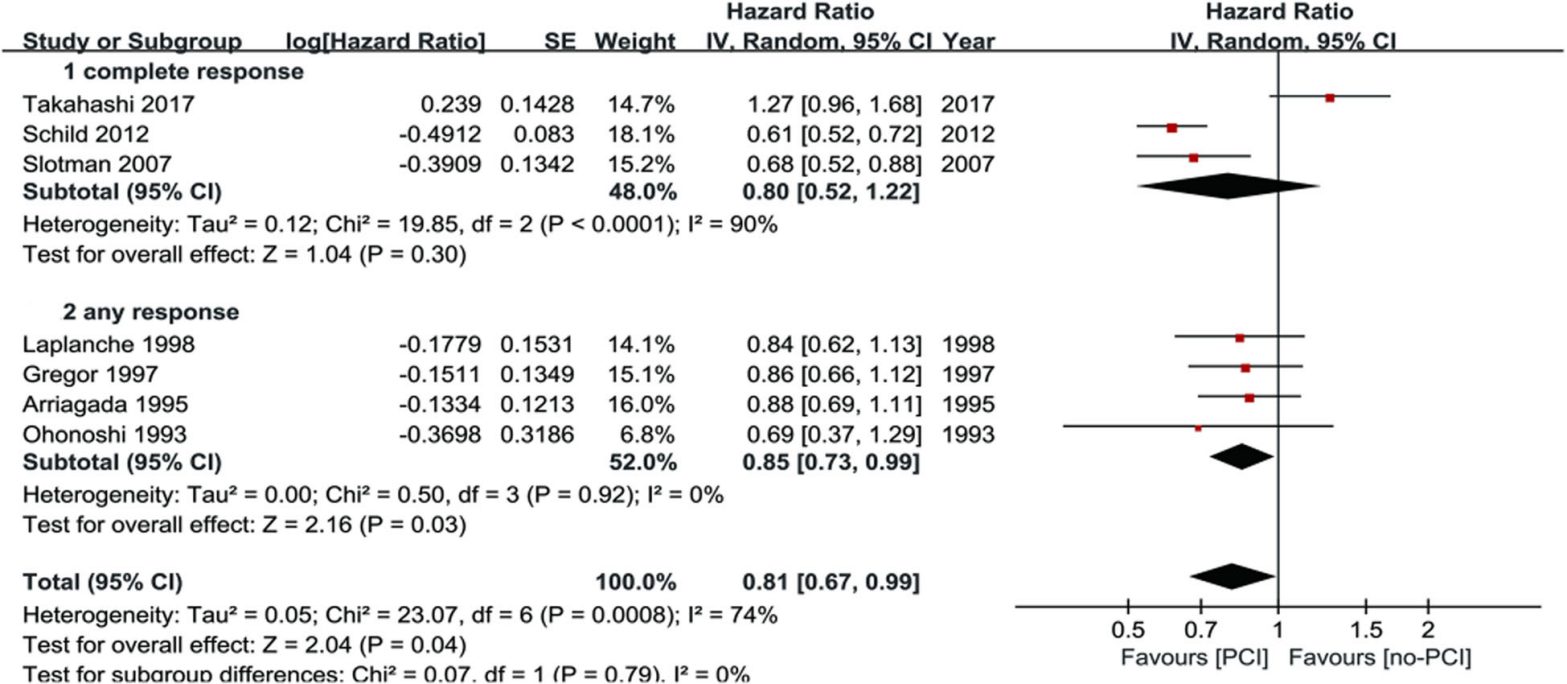
Subgroup analysis for OS according to response to initial chemotherapy

**Fig. 6:**
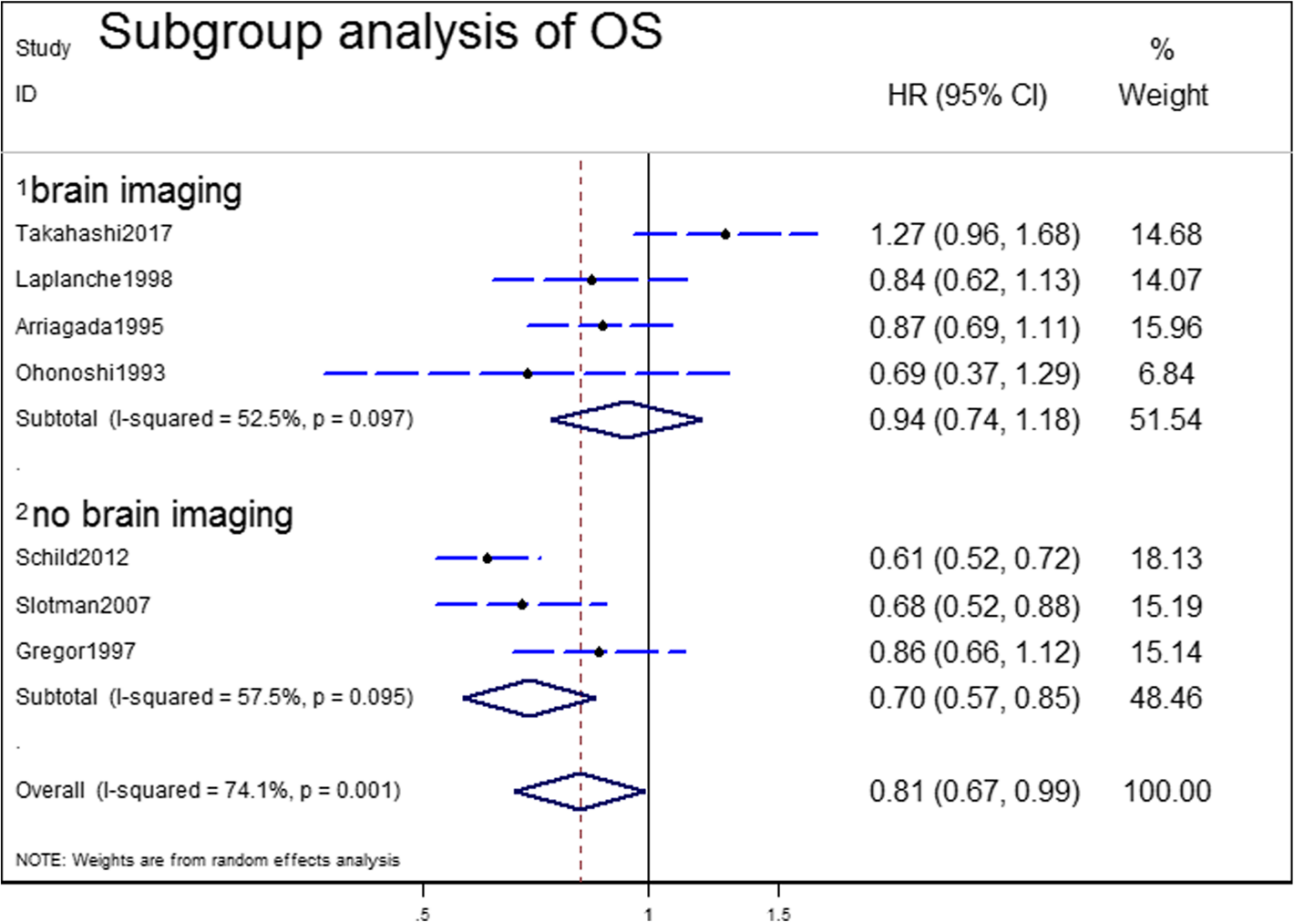
Subgroup analysis for OS according to brain imaging

Subgroup analyses were carried out to explore the heterogeneity in the analysis of OS. Subgroup analyses for OS were performed according to the use of brain imaging after initial CRT, extent of disease and response to initial chemotherapy for patients with SCLC (Table 2). In the three predefined subgroup analyses, the treatment effects were similar between the subgroups by extent of disease (Fig. 4) and response to initial CRT (Fig. 5). The differences in treatment effects in these subgroups were not statistically significant (extent of disease, *P* = 0.67; response to initial chemotherapy, *P* = 0.76). However, the subgroup result by brain scan after initial CRT appeared to be discordant: trials that enrolled patients with brain CT/MRI after initial CRT showed no OS benefit with PCI (HR = 0.94; 95% CI; 0.74–1.18). However, the trials without brain CT/MRI after initial CRT demonstrated significant OS benefit (HR = 0.70; 95% CI: 0.57–0.85) (Fig. 6). The brain imaging after initial CRT might explain the heterogeneity between the trials since the subgroup difference reached the level of statistical significance (*P* = 0.05).

**Table 2:** Subgroup analyses of overall survival

Subgroups	N studies	Pooled HR	95% CI	P value (Heterogeneity between subgroups)
Brain imaging after initial CRT	4	0.94	0.74–1.18	0.0005
No brain imaging after initial CRT	3	0.70	0.57–0.85	
Limited stage	5	0.82	0.71–0.94	0.67
Extensive stage	6	0.76	0.55–1.04	
Any response to initial CRT	3	0.85	0.73–0.99	0.76
Complete response to initial CRT	4	0.80	0.52–1.22	

*HR* hazard radio, *CRT* chemoradiotherapy, *CI* confidence interval, *N* number

Note: this table includes 5 limited stage studies and 6 extensive stage studies, because some trials included in this meta-analysis enrolled both limited and extensive stage small-cell lung cancer and we identified the HR values and pooled them with the individual subgroups

## Discussion

Our present meta-analysis pooled 7 RCTs that evaluated the role of PCI in 2114 patients with SCLC. Interestingly, two RCTs that both studied PCI in patients with ES-SCLC reported opposite outcomes in terms of OS. In general, our meta-analysis revealed a positive role of PCI in improving survival and reducing the risk of BM. However, in subgroup analyses of OS, we found the pooled positive outcome was rather questionable.

Most of the randomized trials had showed a significant decrease of BM incidence; however, none of them individually could demonstrate a significant improvement in OS. Meert et al. [23] revealed positive role of PCI in BM and OS in patients in CR after chemotherapy. More recently, the retrospective study by Patel et al. [24] that involved almost 8000 patients supported the results, with a significant improvement in both overall and cause-specific survival in favor of PCI. However, these positive results were questioned by the most recently published Japanese trial [12]. This recent study found PCI had no benefit in prolonging OS in patients with a confirmed absence of BM when patients received periodic MRI examination during follow-up (HR = 1.27; 95% CI, 0.96–1.68; *P* = 0.094). According to our meta-analysis, the pooled HR showed a slight OS benefit with PCI (HR = 0.81). However, the heterogeneity was high (I^2^ = 74.1%, *P* = 0.001). Thus, we carried out subgroup analyses to explore the heterogeneity in OS according to three aspects: extent of disease, response of initial chemotherapy and the use of brain imaging after initial CRT. The benefit was consistent among subgroups defined according to the extent of disease (*P* = 0.67*)* and response of initial chemotherapy (*P* = 0.76). However, the third subgroup, divided by the use of brain imaging after initial CRT, appeared to be discordant: the subgroup of brain imaging after initial CRT showed no OS improvement with PCI (HR = 0.94; 95% CI; 0.74–1.18) while the subgroup without brain imaging demonstrated an OS benefit (HR = 0.70; 95% CI: 0.57–0.85). This outcome was partly in concordance with the result of Takahashi’s group [12]. However, it is difficult to explain why the group without brain imaging achieved favorable HR. We speculate that the favorable pooled HR may somewhat be due to the recruitment of asymptomatic patients. In other words, trials that did not perform brain imaging after initial CRT might have included a substantial number of patients who already had BM, and asymptomatic BM patients had a worse prognosis. According to Hochstenbag et al. [25], asymptomatic BM was present in about 15% of patients with SCLC at diagnosis. Further, Manapov et al. [26] revealed that 32.5% of patients with LS-SCLC suffered relapse with BM immediately before PCI. The presented treatment for pre-PCI patients with detected BM consisted of either whole-brain radiation alone (WBR) with 3.0 Gy fractions to a total dose of 30 Gy or WBR with 2.0 Gy fractions to a total dose of 40 Gy as a part of second-line CRT with topotecan. Therefore, compared with observation, this subset of asymptomatic BM patients could benefit from the commonly used PCI regimen of 2.5 Gy fractions to a total dose of 25 Gy. As a result, trials that did not perform brain imaging after initial CRT showed a favorable pooled HR of OS.

Among the trials that enrolled patients with imaging proof of no BM, our results showed that these patients benefited little from PCI. Of note, though the pooled HR of this subset of patients was unfavorable, these patients actually had longer survival time. We checked the median survival time of the patients in the 7 trials. In three trials that only enrolled ES-SCLC patients, the median survival times (months) for the PCT group and not PCI group were as follows: 11.6 vs. 13.7, 9.6 vs. 7.9 and 6.7 vs. 5.4, respectively. Among these trials, only the Japanese trial performed brain MRI after initial CRT and before enrollment and reported longer median survival times (11.6 and 13.7) than the other two trials. The above findings were supported by recent retrospective studies of PCI in patients with ES-SCLC [27, 28]. Most of the patients in these recent studies had brain imaging before PCI and the median survival time was similar to the outcome of Japanese trial. The longer median survival time in the trials with brain imaging again affirmed the assumption that a substantial part of patients were enrolled in trials without brain imaging. Therefore, to analyze the efficacy of PCI more accurately, standardized cranial MRI should be taken into consideration in future trials.

In addition to the efficacy of PCI, it is essential to discuss its toxicity. Usually, toxicity of PCI is defined as acute and long-term according to the 3-month cut-off point. Acute toxicity is generally manageable and consists of mostly alopecia, headache, fatigue, nausea and vomiting [29, 30]. Long-term sequelae such as severe memory loss, intellectual impairment or even dementia and ataxia have been reported in several studies and attributed to PCI [18–20, 29, 31–34]. A pooled analysis of the Radiation Therapy Oncology Group (RTOG) randomized trials 0212 and 0214 had shown that PCI was associated with a higher rate of decline in tested and self-reported cognitive functioning [32, 35, 36]. In addition, many confounding factors, such as age and the toxicity of anticancer drugs, may add to the intolerance of neurotoxicity of PCI. Most recently, Farooqi et al. [33] found that the risk of neurotoxicity and neurocognitive decline was greater in elderly patients and those with vascular comorbidities after PCI. In this meta-analysis, we were unable to pool the incidence of toxicity because of limited data. Three trials [11, 18, 20] reported acute reactions and all three found more grade 3 or worse adverse events in the PCI group. Four trials reported late toxicity relating to PCI. Ohonoshi et al. found that late neurologic toxicity was infrequent; only one patient developed a mild deterioration among seven long-term disease-free survivors in the PC1 group. Arriagada et al. [19] reported that the 2-year rates of abnormalities as indicated by CT scans of the brain were 21 and 27% (relative risk = 1.48; *P* = 0.60), respectively. According to Gregor et al. [20], the proportions of patients showing long-term impairment in each test were substantial but similar in the PCI and observation groups. No significant difference was found between the study groups in role functioning (*P* = 0.17), cognitive functioning (*P* = 0.07) or emotional functioning (*P* = 0.18). An analysis of patterns of care in the USA [37] reported a high adherence to guidelines; almost 98% of radiation oncologists recommended PCI for patients with ES-SCLC. Considering the increasing risk of toxicity together with the wide implementation of PCI, a critical re-evaluation of PCI is urgent to determine the appropriate management of SCLC patients. More clinical trials focusing on the analyses of late toxicity are needed in the future.

Several systematic reviews and meta-analyses [10, 38–41] regarding the role of PCI for SCLC survival outcomes have been published. Compared with earlier meta-analyses [23, 38, 39], we set more strict inclusion criteria and excluded trials that didn’t report PCI. Besides, we included several recent trials in our analysis. Two meta-analysis regarding this topic were published this year. Maeng et al. [40] focused on the role of PCI in patients with ES-SCLC. The aim of their study was to perform a systematic review and meta-analysis to determine the role of PCI in patients with ES-SCLC who received PCI. Yang et al. [41] analyzed the BM risk in p-stage I patients without PCI and they only pooled retrospective studies which are not as convincing as random trials. The present meta-analysis has several limitations that merit consideration. First, the significant statistical heterogeneity in the OS meta-analysis could not be fully explained. We were unable to explain why decreased BM come along with unfavorable OS outcome. Second, long-term neurotoxicity could not be addressed in this meta-analysis because neuropsychological evaluation was performed in only two of the trials [12, 19]. Finally, we did not rule out the risk of bias in individual studies, as the number of included articles was less than 10.

## Conclusions

Overall, our meta-analysis reveals that PCI improves OS and significantly decreases BM incidence in SCLC compared with observation. Nonetheless, for patients with confirmed absence of BM by brain imaging, considering the safety of patients, there is insufficient evidence to incorporate PCI into clinical practice. Further RCTs are warranted to verify the role of PCI in screened SCLC patients with contrast-enhanced cranial MRI.

## Additional files

Additional file 1: **Table S1.** Jadad Scoress. (DOCX 14 kb)

Additional file 2: **Figure S1.** The possibility of publication bias across studies was assessed using (a) funnel plots and (b) Begg’s test funnel plot. (DOCX 2790 kb)

## Abbreviations

BM: Brain metastasis
CRT: Chemoradiotherapy
ES-SCLC: Extensive stage small cell lung cancer
HR: Hazard ratio
LS-SCLC: Limited stage small cell lung cancer
OS: Overall survival
PCI: Prophylactic cranial irradiation
RCT: Randomized controlled trial
SCLC: Small cell lung cancer
