# Supplementary material for: Prophylactic cranial irradiation in small cell lung cancer: a systematic review and meta-analysis
Source: BMC Cancer. 2019 Jan 21;19:95. doi: 10.1186/s12885-018-5251-3 (PMC6341615; doi:10.1186/s12885-018-5251-3)
Supplement: Supplementary file 1 — Table S1. Jadad Scoress. (DOCX 14 kb) [file 12885_2018_5251_MOESM1_ESM.docx]

Additional file 1 Table S1: **Jadad Scores**

| **Study** | **Quality Assessment** | | | | |
| --- | --- | --- | --- | --- | --- |
|  | **Randomization** | **Allocation Concealment** | **Double Blinding** | **Withdrawals and dropouts** | **Total** |
| Takahashi et al, 2017 | 2 | 2 | 0 | 1 | 5 |
| Schild et al, 2012 | 1 | 1 | 1 | 1 | 4 |
| Slotman et al, 2007 | 1 | 2 | 1 | 1 | 5 |
| Laplanche et al, 1998 | 2 | 1 | 1 | 1 | 5 |
| Gregor et al, 1997 | 1 | 1 | 1 | 1 | 4 |
| Arriagada et al, 1995 | 1 | 1 | 1 | 1 | 4 |
| Ohonoshi et al, 1993 | 1 | 1 | 1 | 1 | 4 |
