# Supplementary figures and images for: Prophylactic cranial irradiation in small cell lung cancer: a systematic review and meta-analysis
Source: BMC Cancer. 2019 Jan 21;19:95. doi: 10.1186/s12885-018-5251-3 (PMC6341615; doi:10.1186/s12885-018-5251-3)

Additional file 2: Figure S1a


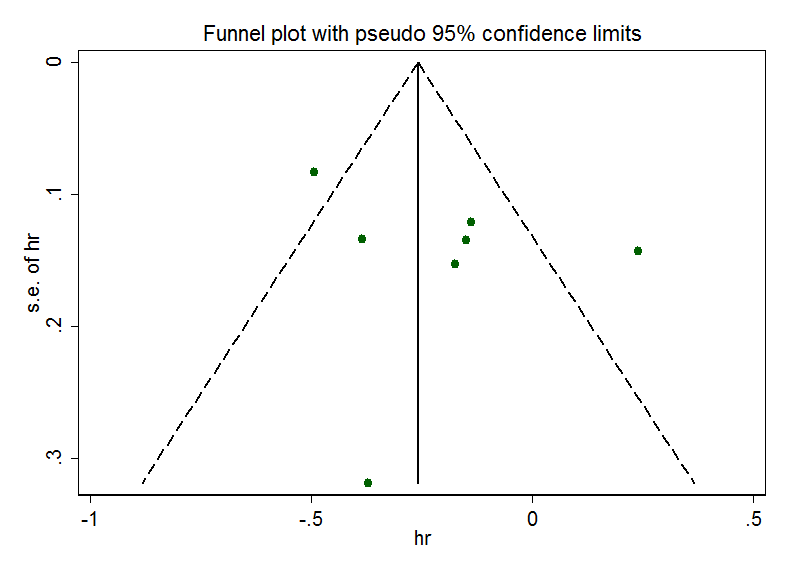


Figure S1b


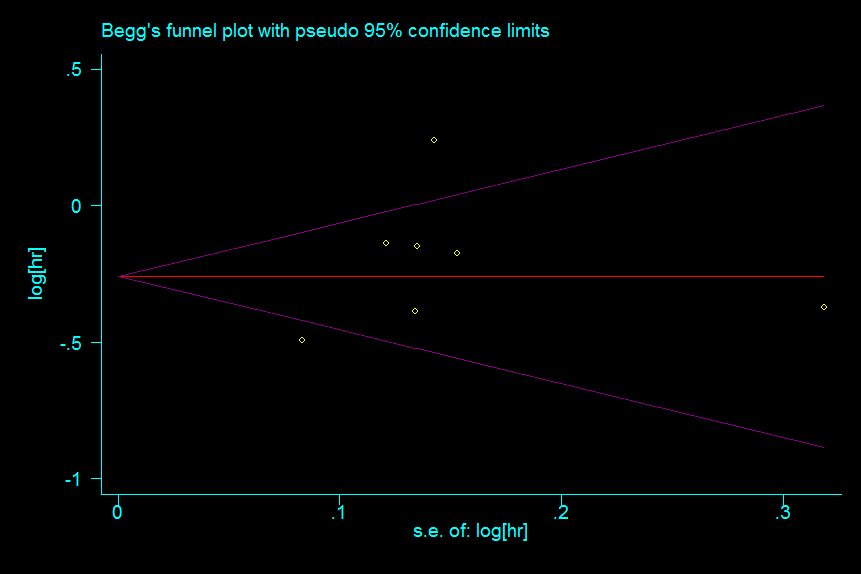

Supplement: Supplementary file 2 — Figure S1. The possibility of publication bias across studies was assessed using (a) funnel plots and (b) Begg’s test funnel plot. (DOCX 2790 kb) [file 12885_2018_5251_MOESM2_ESM.docx]
